# Supplementary material for: IntegrateALL: An end‐to‐end RNA‐seq analysis pipeline for multilevel data extraction and interpretable subtype classification in B‐precursor ALL
Source: Hemasphere. 2026 Apr 16;10(4):e70366. doi: 10.1002/hem3.70366 (PMC13084704; doi:10.1002/hem3.70366)
Supplement: Supplementary file 1 — Additional supporting information can be found in the online version of this article. Wolgast N 2026 Supplementary Figures. [file HEM3-10-e70366-s001.pdf]

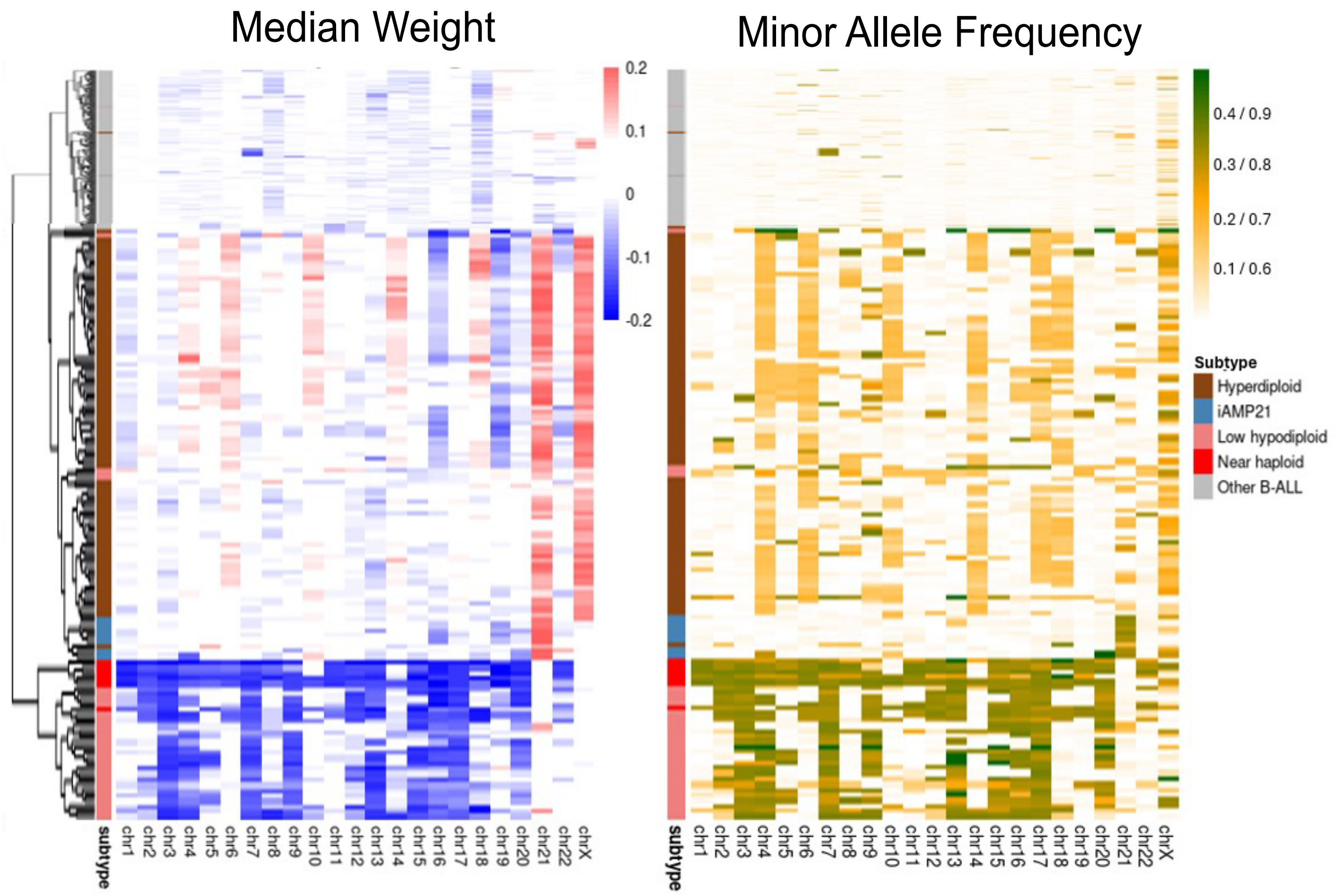

Supplementary Figure S1: Heatmaps of RNASeqCNV-derived data (median weight and minor allele frequency) across the training cohort (n=395), annotated by subtype. Chromosomal gains and losses derived from normalized read counts are shown in red and blue respectively. To visualize the corresponding minor allele frequency values (MAF), we analyzed the frequency distribution of MAF values to identify the maximum MAF for each chromosome and sample. These maxima were transformed to mode of 0.5 minus MAF maximum, centering even chromosome numbers (diploidy, tetraploidy) around 0 (white), triploidy around 0.2 (yellow) and monosomy / LOH around 0.4 (green). Samples were clustered according to Median Weights using Ward.D2. Cases with ‘other B-ALL’ karyotypes are shown in a compressed view to improve visibility of aneuploid cases.

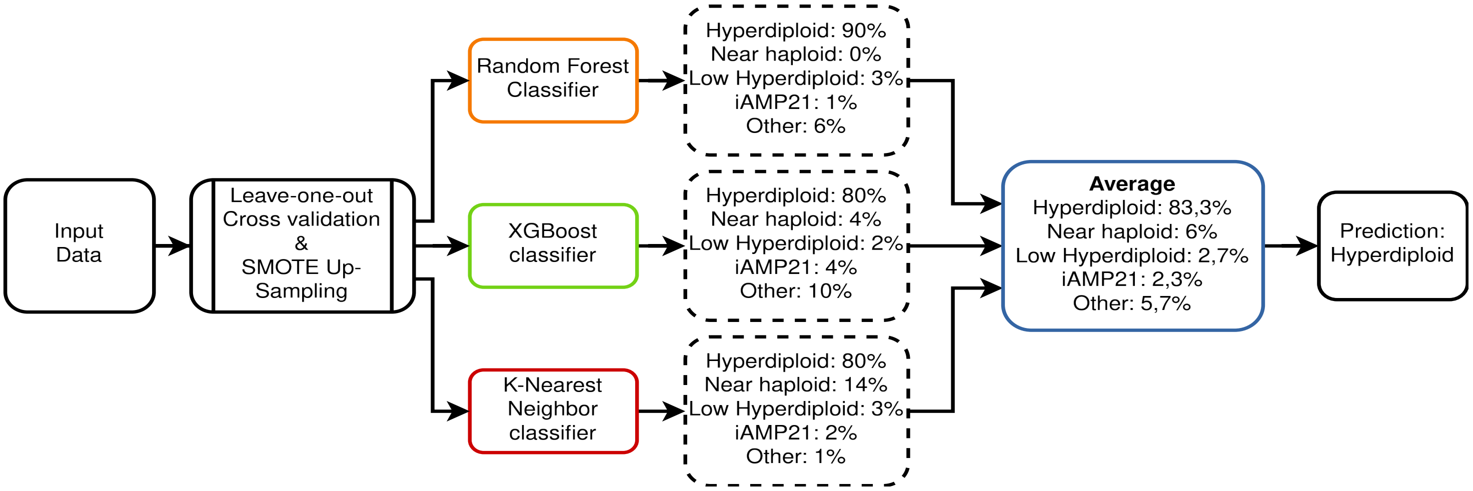

Supplementary Figure S2: Workflow of the KaryALL classifier. During training, input features derived from RNASeqCNV were processed using SMOTE up-sampling and evaluated with leave-one-out cross-validation. The final model is implemented as a soft-voting ensemble combining Random Forest, XGBoost, and k-NN classifiers.

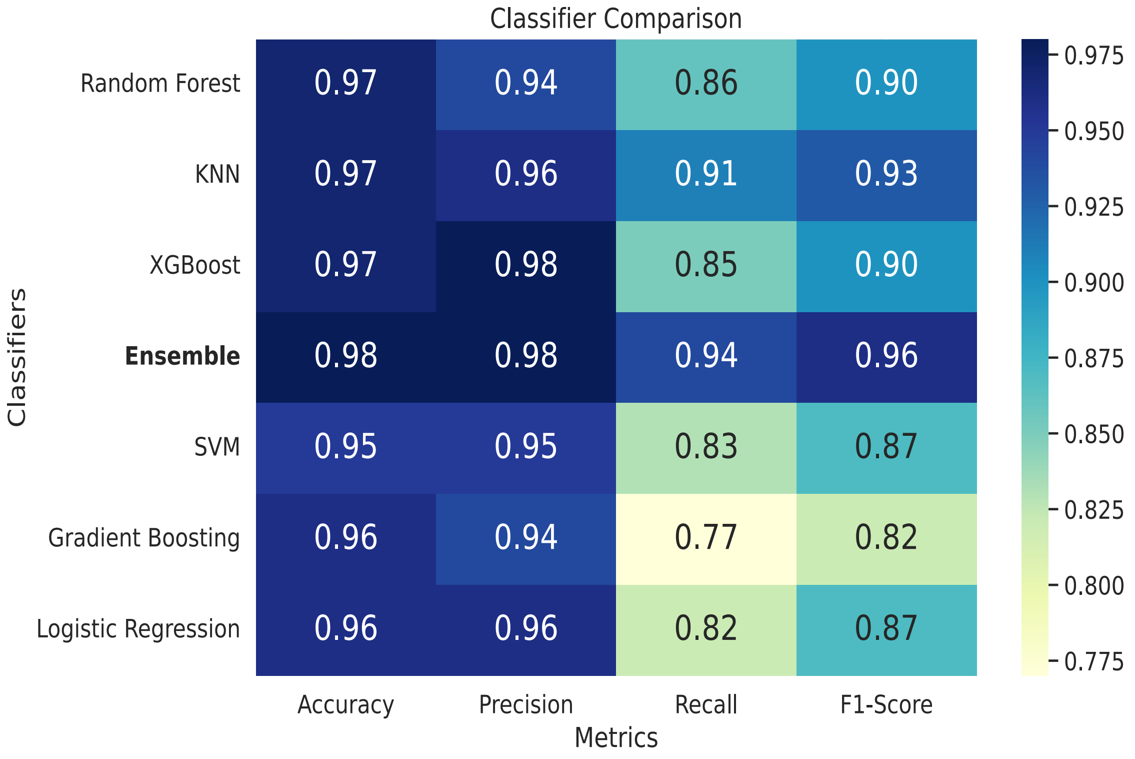

Supplementary Figure S3: Classifier comparison on n=395 samples of the training set. Performance comparison of six single machine learning classifiers and the ensemble model using accuracy, precision, recall, and F1-score; the ensemble outperforms all individual models.
The corresponding classification results of the ensemble classifier are shown in Main Figure 2D,E.

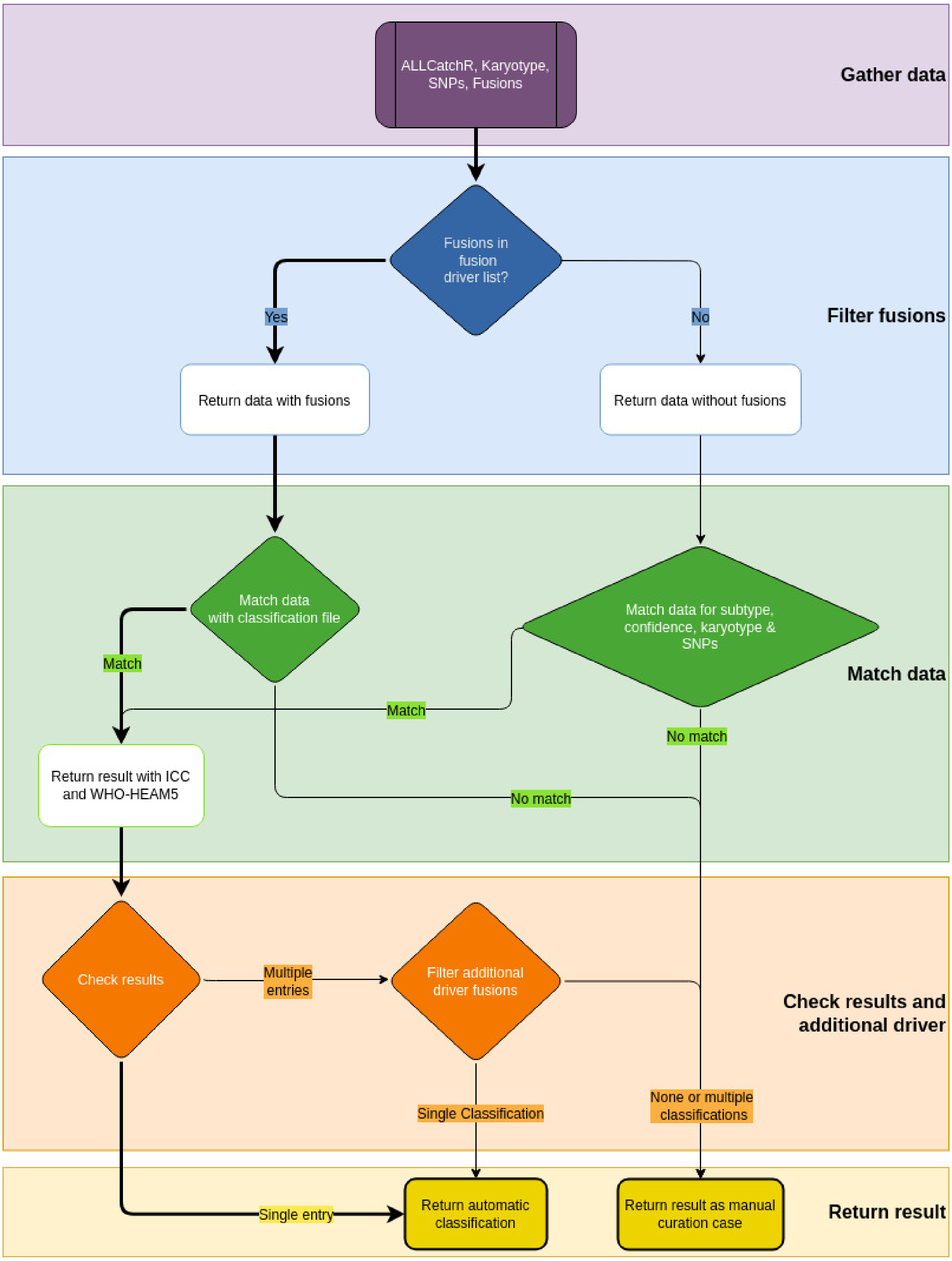

Supplementary Figure S4: Schematic overview of the IntegrateALL classification algorithm to assign molecular subtypes based on multi-layered gene expression data and inferred drivers. The flowchart is organized into five color-coded levels, labeled on the right margin: Gather data (purple), Filter fusions (blue), Match data (green), Check results and additional drivers (orange), and Return result (yellow). Data inputs include fusion calls, gene expression profiles, SNV hotspots, and RNASeqCNV-based virtual karyotypes. Fusion results are first filtered against a curated list of subtype-defining drivers. Fusion-positive samples are matched to a classification reference to assign an ICC and WHO-HAEM5 subtype; unmatched cases proceed to further evaluation. Fusion-negative samples are assessed for concordance between expression-based subtype, prediction confidence, karyotype, and SNVs.
If predefined criteria are met, diagnostic labels are assigned; otherwise, the case is flagged for manual curation. In the decision phase preceding classification, the algorithm determines whether a unique classification can be made. If multiple potential classifications or additional drivers are detected, further filtering is applied. The final decision returns either an automatic classification or flags the case for manual review.

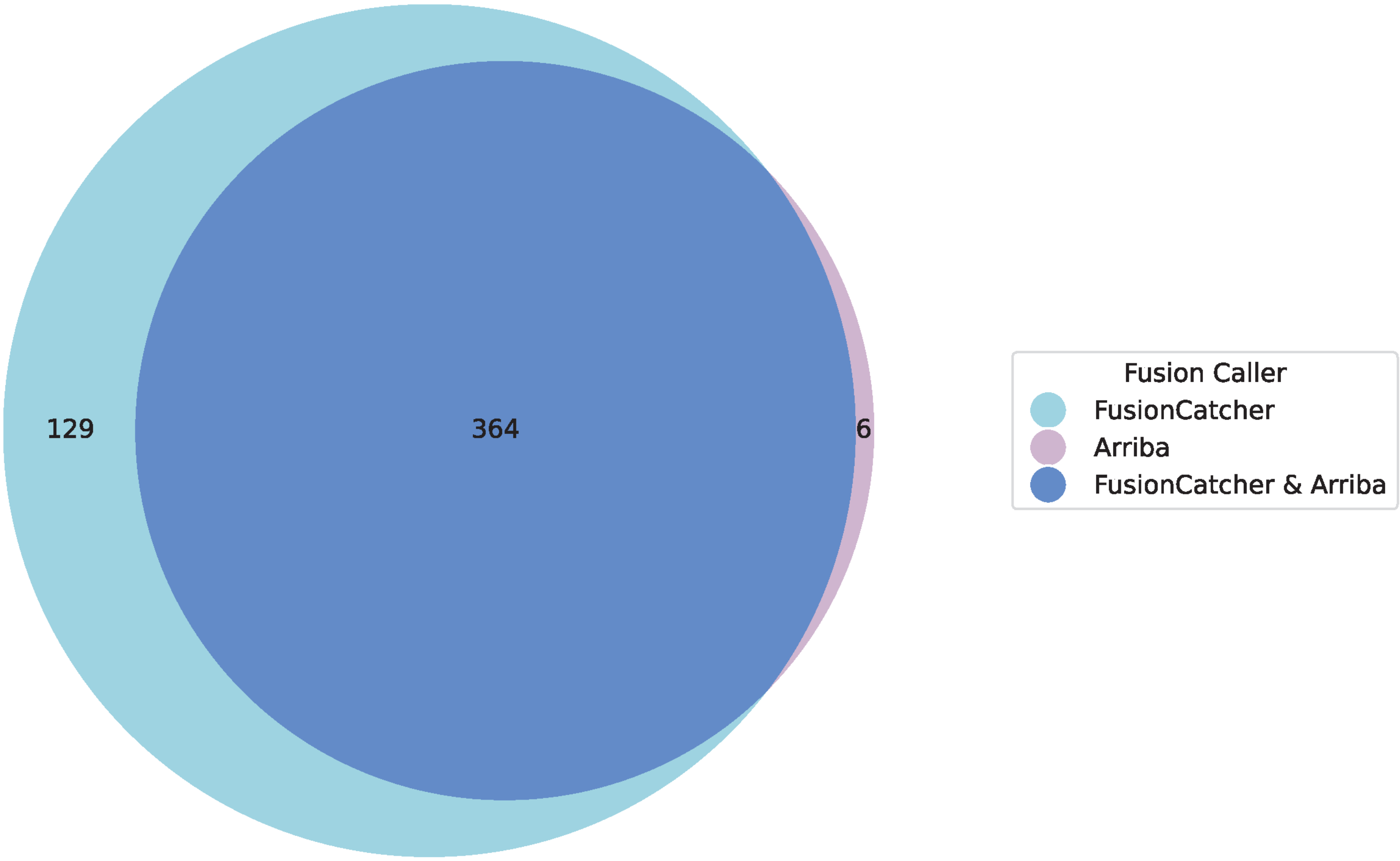

Supplementary Figure S5: Comparison of fusion calls by FusionCatcher and Arriba. Fusion calls from both callers mapping to our B-ALL driver reference list across the primary cohort of 774 samples are shown. From a total of 499 fusion calls, 72.9% were identified by both callers, 25.9% were exclusive to FusionCatcher and 1.20% were exclusive to Arriba. Notably, certain fusions such as *UBTF*::*ATXN7L3* and *IGH*::*DUX4* were preferentially identified by FusionCatcher. The full list of fusion calls is provided in Supplementary Table S4.

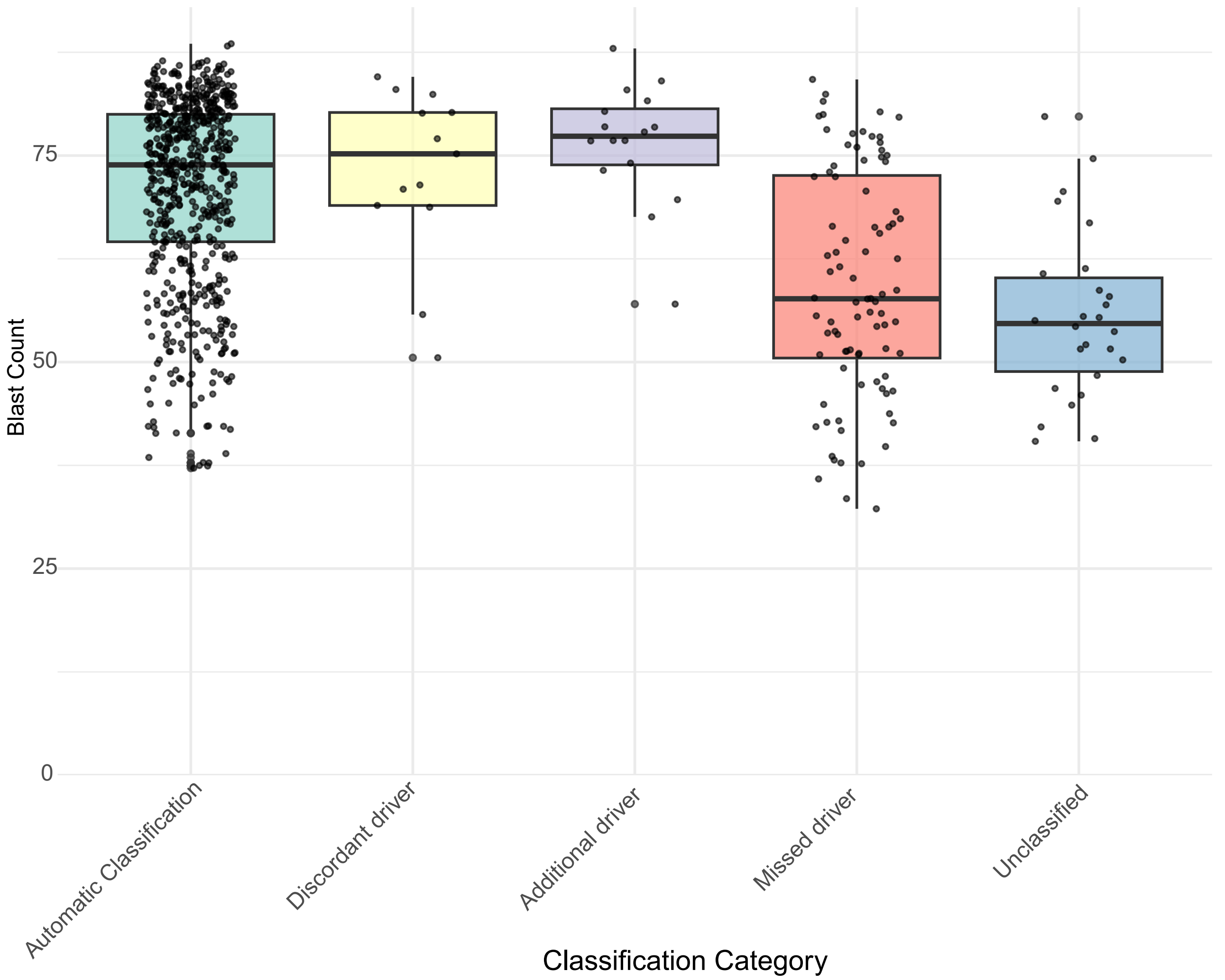

Supplementary Figure S6: Comparison of blast count predictions across different driver constellations. Blast count infiltration was predicted by ALLCatchR. Predicted blast counts were compared between samples with automated subtype allocation and different categories of manual curation.

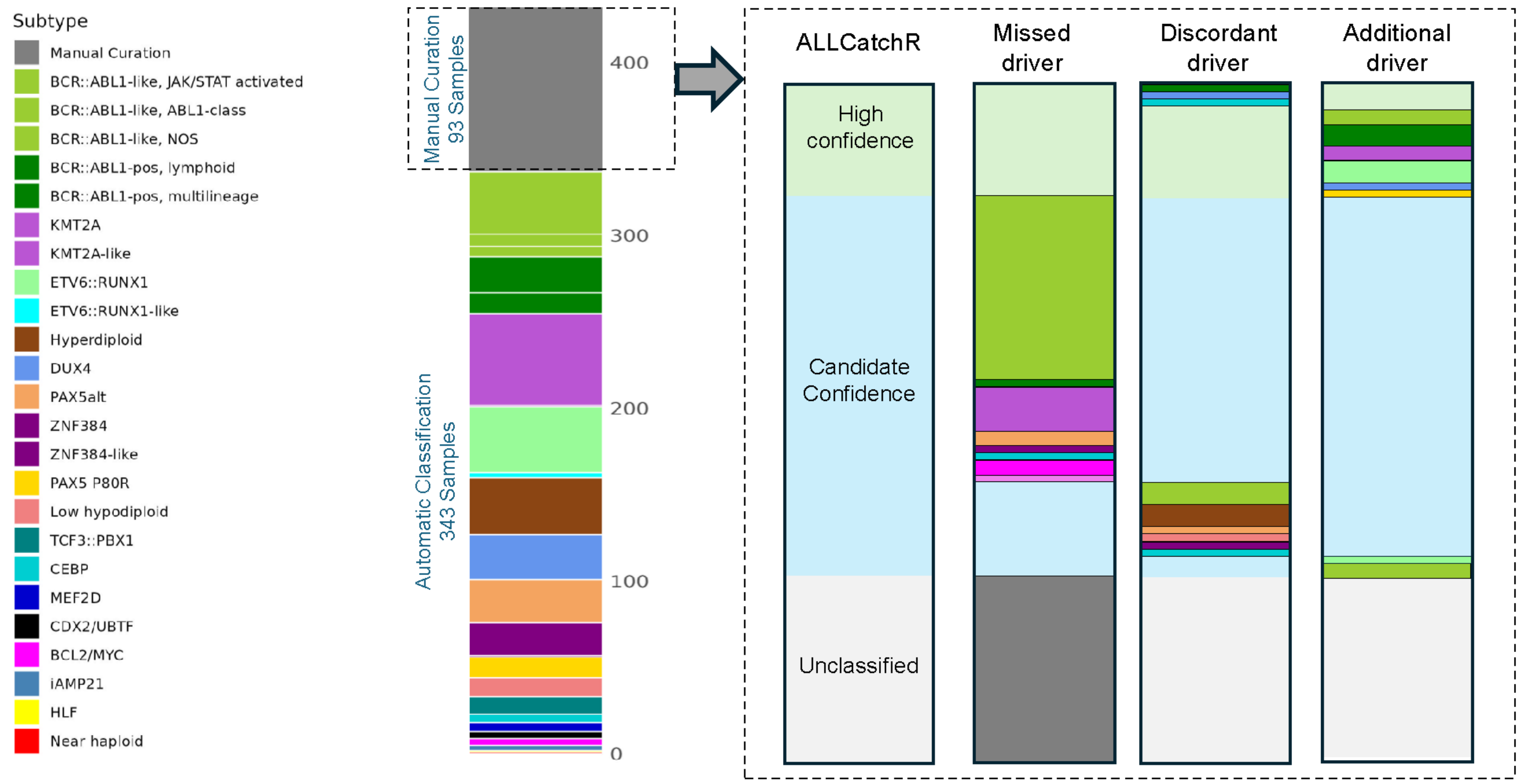

Supplementary Figure S7: Testing of IntegrateALL in external cohorts. We processed through IntegrateALL n=436 B-ALL samples from own sequencing during routine diagnostics (Munich Leukemia Lab, MLL; n=105) and an independent second research GMALL cohort (n=98) as well as published cases (Autry RJ et al. Nat Cancer 2020; n=233). In total n=16/105 (15.2%; MLL), n=16/98 (16.3%; 2nd GMALL cohort) and n=61/233 (n=26.2%; Autry RJ et al.) were flagged for manual curation while the remaining cases (78.7% across all three cohorts) were automatically classified. Results of manual curation are shown similar to Main Figure 5B.

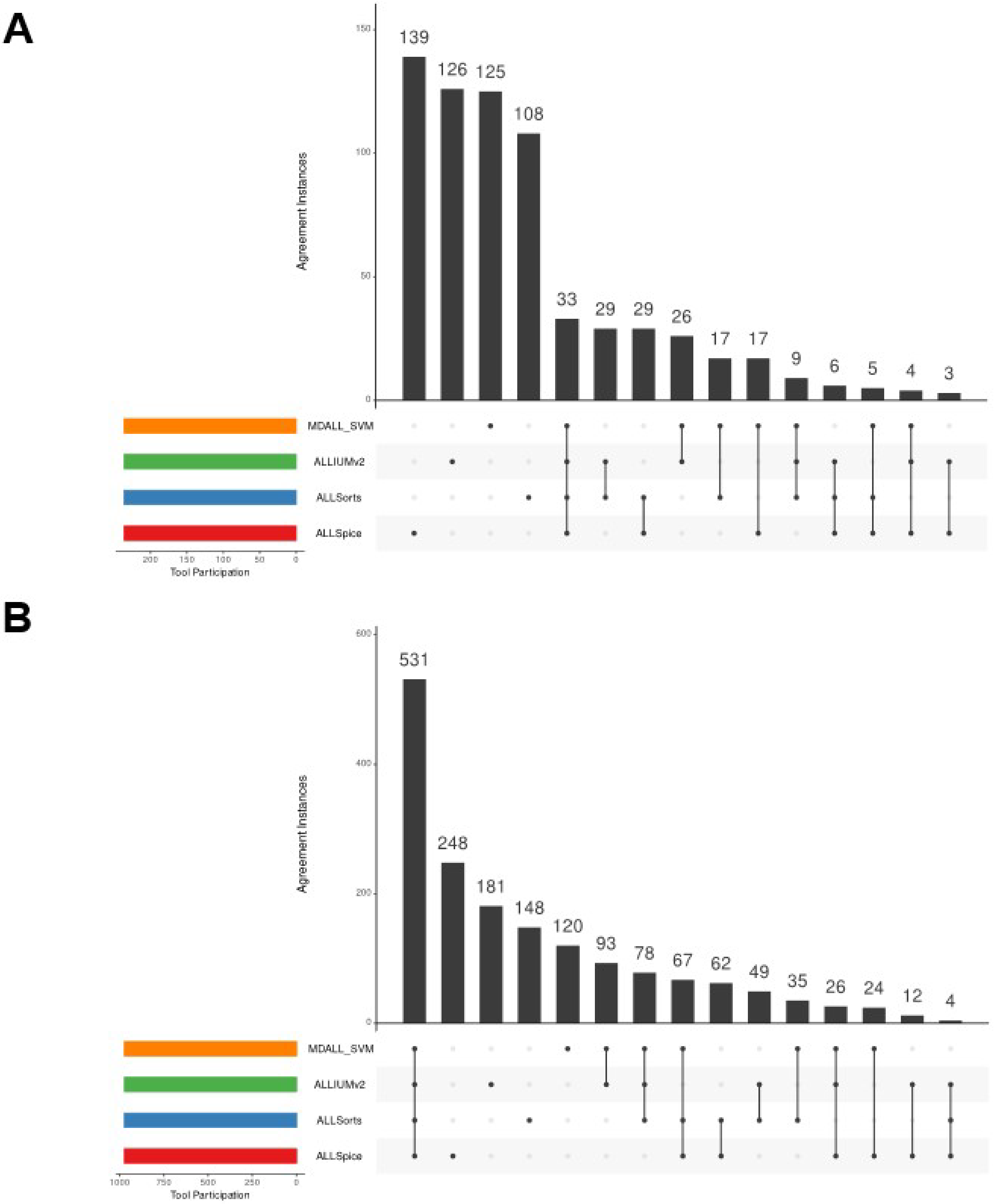

Supplementary Figure S8: Inter-tool concordance analysis of B-ALL subtype classification. UpSet plots comparing prediction concordance across four B-ALL classification tools (ALLSpice, ALLSorts, ALLIUMv2, and MDALL-SVM). Each vertical bar represents a unique tool combination with its prediction frequency shown above. Connected dots below bars indicate which tools contributed to that prediction set. (A) Analysis of 236 manually curated samples demonstrating increased classification complexity and tool discordance (2.86 predictions per sample). Single-dot intersections represent cases where only one tool made a unique prediction with no concordance from other tools, highlighting classification uncertainty. (B) Analysis of 974 automatically classified samples showing higher inter-tool agreement (1.72 predictions per sample).
